# Discovery of a novel glucose metabolism in cancer: The role of endoplasmic reticulum beyond glycolysis and pentose phosphate shunt

**DOI:** 10.1038/srep25092

**Published:** 2016-04-28

**Authors:** Cecilia Marini, Silvia Ravera, Ambra Buschiazzo, Giovanna Bianchi, Anna Maria Orengo, Silvia Bruno, Gianluca Bottoni, Laura Emionite, Fabio Pastorino, Elena Monteverde, Lucia Garaboldi, Roberto Martella, Barbara Salani, Davide Maggi, Mirco Ponzoni, Franco Fais, Lizzia Raffaghello, Gianmario Sambuceti

**Affiliations:** 1 CNR Institute of Molecular Bioimaging and Physiology (IBFM), Milan, Section of Genoa, Genoa, Italy; 2 Nuclear Medicine Unit, Department of Health Sciences, University of Genoa and IRCCS AOU San Martino-IST, Genoa, Italy; 3 Stem Cell Center, IRCCS G. Gaslini, Genoa, Italy; 4 Laboratorio di Oncologia, IRCCS G. Gaslini, Genoa, Italy; 5 Department of Experimental Medicine, University of Genoa, Genoa, Italy; 6 Animal facility, IRCCS AOU San Martino-IST, Genoa, Italy; 7 Department of Internal Medicine, University of Genoa and IRCCS AOU San Martino-IST, Genoa, Italy; 8 Molecular Pathology, IRCCS AOU San Martino-IST, Genoa, Italy

## Abstract

Cancer metabolism is characterized by an accelerated glycolytic rate facing reduced activity of oxidative phosphorylation. This “Warburg effect” represents a standard to diagnose and monitor tumor aggressiveness with ^18^F-fluorodeoxyglucose whose uptake is currently regarded as an accurate index of total glucose consumption. Studying cancer metabolic response to respiratory chain inhibition by metformin, we repeatedly observed a reduction of tracer uptake facing a marked increase in glucose consumption. This puzzling discordance brought us to discover that ^18^F-fluorodeoxyglucose preferentially accumulates within endoplasmic reticulum by exploiting the catalytic function of hexose-6-phosphate-dehydrogenase. Silencing enzyme expression and activity decreased both tracer uptake and glucose consumption, caused severe energy depletion and decreased NADPH content without altering mitochondrial function. These data document the existence of an unknown glucose metabolism triggered by hexose-6-phosphate-dehydrogenase within endoplasmic reticulum of cancer cells. Besides its basic relevance, this finding can improve clinical cancer diagnosis and might represent potential target for therapy.

In most solid cancers, the high needs of ATP and macromolecules for the rapidly growing biomass result in accelerated glycolysis facing relatively low rates of Krebs cycle and oxidative phosphorylation (OXPHOS)^1^. Although exploiting this “Warburg effect”^2^ already became a clinical standard to diagnose and monitor cancer aggressiveness by ^18^F-fluorodeoxyglucose (FDG) imaging, its underlying mechanisms remain elusive and, in particular, an open discussion still exists about the role of mitochondrial injury^3^.

Addressing this uncertainty, we were evaluating cancer metabolic response to OXPHOS inhibition by metformin (MTF). In our starting hypothesis, the severe respiratory impairment^4^,^5^ should have triggered a “Pasteur effect” enhancing glycolytic flux^6^. By contrast, we repeatedly observed a dose-dependent reduction in FDG retention in response to MTF that preceded a decrease in proliferation rate in different cancer cell lines, both *in vitro* and *in vivo*^7^,^8^,^9^.

According to a largely accepted kinetic model, uptake of FDG and 2-deoxyglucose (2DG) depicts overall glucose consumption because both analogues enter the cytosol through GLUT carriers, are phosphorylated by hexokinases (HK) and accumulate within the cytosol being false substrates for downstream enzymes channeling glucose-6-phosphate (G6P) to glycolysis and pentose phosphate pathway (PPP)^10^,^11^,^12^,^13^. The decrease in FDG retention under MTF was thus interpreted as an index of reduced glucose consumption despite the OXPHOS impairment. This finding at least partially agreed with studies on cancer metabolism using the same radionuclide approach^14^,^15^. However, it profoundly disagreed with the marked increase in glycolytic flux documented *in vitro* by direct measurements of glucose consumption and lactate release under comparable MTF doses^14^,^16^.

Investigating the divergent MTF effects on FDG retention and glucose consumption brought us to discover that tumor FDG uptake is largely independent from overall glucose utilization and tracks a novel monosaccharide metabolism that is triggered by hexose-6-phosphate dehydrogenase (H6PD) within the lumen of endoplasmic reticulum (ER), is fueled by glucose at high rates and is strictly related to cancer growth and aggressiveness.

## Results

### Metformin affects cancer metabolism *in vivo*

**Figure 1:**
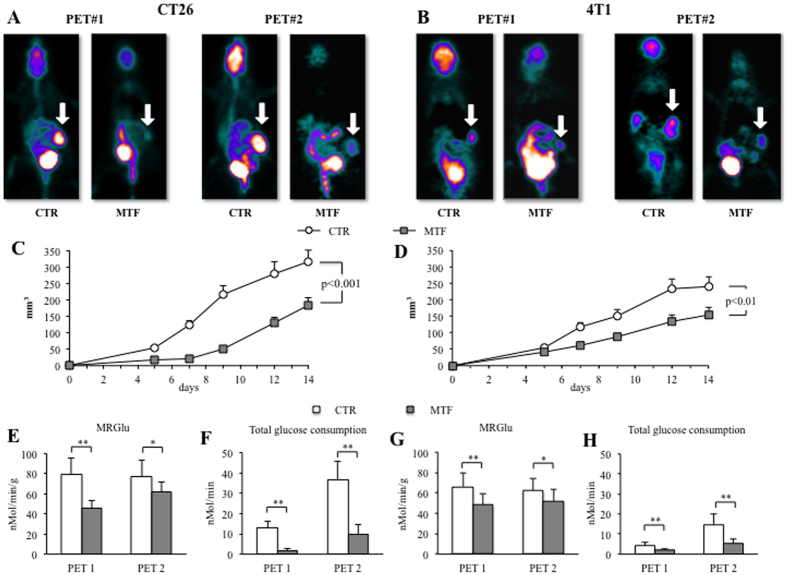
*In vivo* effect of metformin on tumor glucose consumption and cancer growth. CT26 and 4T1 cells were subcutaneously inoculated in the hip of BALB/c mice (200.000 cells/mouse). Treatment with metformin (MTF) (750 mg/Kg die) started 48 hours after tumor implantation and was maintained throughout study duration. Animals were divided into four groups of seven mice each. Imaging was performed at week #1 and week #2 after implantation using a dedicated micro-PET system. Panel (**A**) shows the parametric maps of representative mice untreated (CTR) or under MTF therapy, one week (PET#1) or two weeks (PET#2) after CT26 implantation. The same sequence is reported in panel (**B**) shows for representative mice implanted with 4T1 cells. White arrows indicate the tumor mass. Panels (**C**,**D**) show average tumor volume expressed in mm^3^ in the corresponding groups with untreated and MTF lesions being indicated as white circle and gray squares, respectively. Glucose consumption is represented as average value throughout lesion mass (in nMol x min^−1^ x gr^−1^ panel (**E**) or total disposal in the entire lesion volume (in nMol x min^−1^, panel (**F**) for CT26 group, while Panel (**G**,**H)** follow the same scheme for 4T1 animals. Both PET indexes of cancer glucose consumption were significantly lower in MTF (gray columns) with respect to untreated (white columns) animals. (*=p < 0.05; **=p < 0.01 vs corresponding controls).

In our previous studies, MTF effect on FDG uptake was tested in xenografts of breast cancer in athymic (nu/nu) mice^8^. Since immune/inflammatory pathways may influence biguanide anticancer potential^17^, we extended our evaluation to murine colon (CT26) and breast (4T1) carcinomas, subcutaneously implanted in the hip of immuno-competent BALB/c mice. In agreement with the use of non-diabetic models, body weight and serum glucose levels were not significantly affected at any time by the high drug dosage used (750 mg/kg per day)^8^,^18^ (Supplementary Table 1). Nevertheless, dynamic micro-PET imaging showed a significant and persistent cancer metabolic response to MTF both at weeks #1 and #2 after tumor implantation (Fig. 1A,B). The relevance of this impairment was corroborated by the evident decrease in cancer growth rate during the whole study duration (Fig. 1C,D). Moreover, compartmental analysis of FDG accumulation^19^ documented that MTF markedly decreased both average and total lesion glucose consumption at one and two weeks after implant of both CT26 and 4T1 cancer cells (Fig. 1E,F).

### Different kinetics of FDG and glucose in cancer

**Figure 2:**
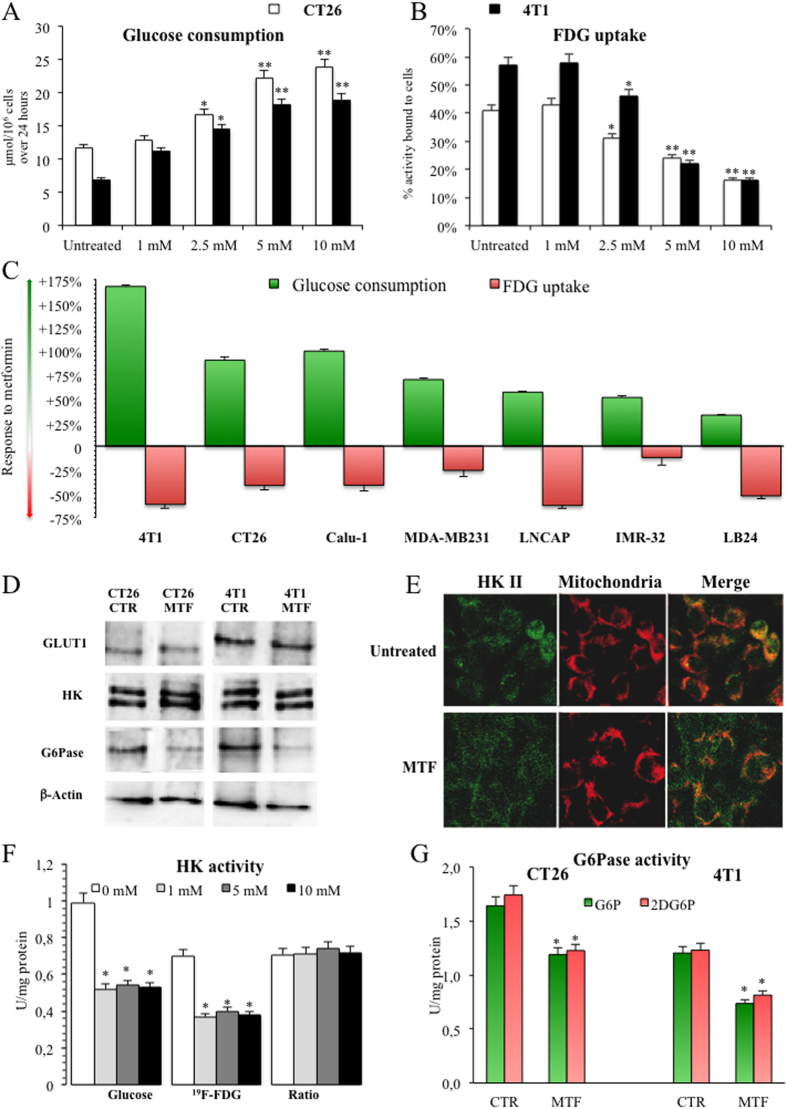
Direct MTF effect on glucose consumption and FDG uptake. Panel (**A**) shows the evident dose-dependent increase in glucose consumption (expressed in μMol/10^6^ cells per day) under MTF treatment in both CT26 (white columns) and 4T1 (black columns) cells. This behavior was paralleled by a progressive and dose-dependent decrease in FDG uptake (expressed as % of total administered radioactivity) in the same cell cultures (Panel **B**) All experiments were performed in triplicate. A similar discrepancy occurred when the experiment was repeated in a broad panel of cancer cell lines using a fixed MTF concentration (5mM). Besides 4T1 CT26, this finding also applied to human models of non-small cell lung cancer (Calu-1), triple negative breast cancer (MDA-MB231) prostate cancer (LNCAP), neuroblastoma (IMR-32) and melanoma (LB24). This opposite response is represented in Panel (**C)** Data are reported as difference (MTF-control) divided by control value for glucose consumption (green columns) and FDG uptake (pink columns). Panel (**D**) displays gels (run under the same experimental conditions for each cell line) reporting the effect of 24 hours exposure to 5 mM MTF on expression of acknowledged determinants of FDG kinetics, i.e. uptake (GLUT1) and entrapment (HKII), in both 4T1 and CT26 cell lines. The drug even decreased G6Pase expression whose availability is warranted to allow FDG6P de-phosphorylation. This inhibition was paralleled by enzyme dislocation out from the mitochondrial membrane (Panel **E**,**F**) shows the moderate decrease of total HK activity in CT26 cell lysates (expressed as U x mg^−1^ of proteins, data for 4T1 are reported in Supplementary Figure 3). However, this response could not explain the divergent responses of glucose and FDG to MTF. In fact, cell lysate affinity ratio for glucose and nonradioactive FDG form ^19^F-FDG remained stable despite increasing drug concentrations. Panel (**G**) displays G6Pase enzyme activity on G6P (green) and 2DG6P (as an analogue of FDG6P, pink) in lysates of both CT26 and 4T1 cell lines (expressed as U × mg^−1^ of proteins). Dephosphorylation rate of both substrates was decreased to a similar degree by 24 hours exposure to 5 mM MTF indicating that drug-induced increase in tracer loss was not caused by a selective enhancement of G6Pase activity on glucose analogues.

The standard PET approach for *in vivo* measurements of glucose metabolism indicated a minor role for immune and inflammatory mechanisms in cancer metabolic response to MTF. We thus hypothesized that the divergent drug effects on glucose consumption and FDG retention reported in the literature^7^,^8^,^14^,^16^, might actually reflect different metabolic fates for the two metabolites. To test this hypothesis, we first planned a series of *in vitro* studies to simultaneously measure FDG uptake and glucose consumption in the same cell culture. Since in our previous experience, MTF action on FDG uptake was independent from glucose concentration, we tested the effect of different drug doses using a constant monosaccharide availability in the culture medium (11.1 mM)^20^. In both CT26 and 4T1 cell lines, MTF markedly increased glucose disappearance from supernatant and profoundly decreased FDG uptake in a dose-dependent fashion (Fig. 2A,B). To determine whether this puzzling discordance is a peculiar feature of studied murine cell lines, we extended this evaluation to a broad panel of human cancers of different origin (lung, breast, prostate, neuroblastoma and melanoma) using the maximal MTF dose devoid of evident cytotoxic effect (5 mM). In naïve cultures, this experiment confirmed the expected, direct relationship between glucose consumption and FDG uptake (Supplementary Figure 1). However, it also confirmed the divergent response to MTF that increased glucose disappearance from culture medium on average to 161 ± 8% of baseline value (range 135–268%), while decreasing FDG uptake on average to 60 ± 5% of corresponding control values (range 88–38%) (Fig. 2C).

Technical analysis of tracer kinetics confirmed the metabolic nature of this paradox. On one side, thin layer chromatography ruled out any breakdown of free ^18^F^−^, documenting that ≥94% of supernatant radioactivity was actually FDG-linked without any difference in migration profile regardless drug presence or absence (Supplementary Figure 2). Similarly, the acknowledged determinants of FDG transport and phosphorylation did not respond to MTF. In fact, drug exposure left unaltered GLUT1 availability^21^,^22^ (Fig. 2D) and did not affect HKII expression (Fig. 2D), although it partially removed the enzyme connection with mitochondrial membrane^7^,^8^,^23^ (Fig. 2E). Total HK activity was actually reduced in cell lysates of both CT26 (Fig. 2F) and 4T1 (Supplementary Figure 3). However, this effect applied to the same extent to glucose and its analogue FDG (in its non-radioactive form ^19^F-FDG) while affinity ratio for the two substrates remained stable at 0.71 ± 0.06 for both cell lines regardless exposure to MTF (Fig. 2F, Supplementary Figure 3).

Therefore, the ratio between cell production rate of G6P and FDG6P remained constant regardless increasing drug concentrations, indicating that trans-membrane transport and entrapment mechanisms could not explain the divergent response of FDG and glucose to MTF.

We thus hypothesized the presence of an escape pathway represented by the possible occurrence of FDG6P de-phosphorylation. The canonical enzyme responsible for this activity is G6P-phosphatase (G6Pase), a complex of multiple proteins anchored to ER lumen whose expression has been mostly characterized in liver, kidney and gut due to their homeostatic role in regulating blood glucose levels^24^,^25^. Actually, FDG6P is a recognized substrate for this enzyme whose activity explains the relatively low sensitivity of PET-FDG in hepatocellular carcinoma as the unique cancer type in which G6Pase has been described so far^26^.

G6Pase activity was well represented in cell lysates of both CT26 and 4T1, was comparable to the one of rat liver homogenates and displayed a similar affinity for G6P and 2DG6P, as an analogue of the radioactive FDG6P (Supplementary Figure 4A,B). However, MTF significantly and equally reduced G6P and 2DG6P dephosphorylation rate in both cell lines (Fig. 2G). This response was not caused by any drug interference on enzyme activity since MTF addition to lysates of untreated cells was ineffective (Supplementary Figure 4A,B); rather, it reflected a decrease in protein expression (Fig. 2D) reproducing in studied cancer cells the mechanism of MTF anti-hyperglycemic action in hepatocytes^27^. Accordingly, cell capability to dephosphorylate FDG6P was actually lowered by MTF, indicating that G6Pase could not account “per se” for the divergent drug actions on tracer retention and glucose consumption.

### Endoplasmic reticulum and FDG uptake in cancer

Altogether, these findings suggested that FDG retention might not represent the simple product of transporter-mediated uptake and subsequent trapping by phosphorylation. Rather, it might reflect the activity of MTF-sensitive metabolic pathways competing with G6Pase for FDG handling. Actually, accumulation of FDG metabolites downstream FDG6P has been already described mostly in normal tissues of non-neuronal origin^28^, although this issue has been scarcely addressed in cancer.

G6Pase is located within the ER lumen^24^,^27^,^29^ in strict connection with another G6P-processing machinery governed by H6PD^30^,^31^. This autosomally-linked enzyme catalyzes the first two reactions of PPP within ER transforming G6P into 6-phospho-gluconolactone/6-phospho-gluconate. Since its activity is remarkably lower with respect to its cytosolic counterpart sex-linked G6PD, H6PD is usually considered as a regulator of the signaling pathway tuning activation-inactivation of steroid hormones. This activity implies the supply of NADPH as a cofactor for the enzymatic function of 11-β−hydroxy-steroido-reductase^32^,^33^. However, its ubiquitous expression and its wide intracellular distribution^31^,^34^ suggest a housekeeping role in ER redox homeostasis.

Our interest in this enzyme was justified by its acknowledged capability to process a large number of substrates beyond G6P^35^. This concept was confirmed by our preliminary experiments in which, differently from G6PD, purified H6PD was able to oxidize not only G6P but also 2DG and 2DG6P (Supplementary Figure 4C,D), suggesting a possible role for this enzyme in FDG6P processing. Actually, H6PD function was evident in both CT26 and 4T1 lines, and its strict association with intracellular membrane^30^,^32^ was confirmed by the need of prolonged cell sonication to extract its full activity in both cell lines (Supplementary Figure 4E).

**Figure 3:**
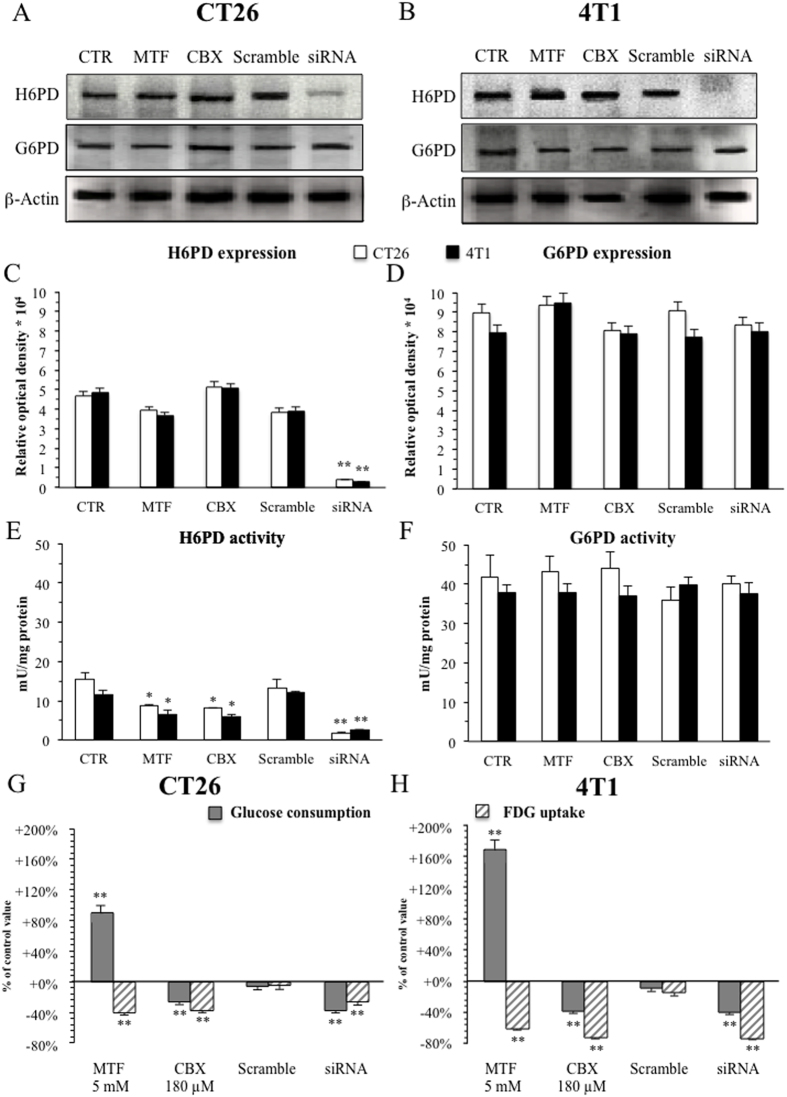
H6PD and glucose metabolism in cancer. Panels (**A,B**) display gels, (run under the same experimental conditions for each cell line), of H6PD and G6PD expression, evaluated with Western blot analysis. Quantitative analysis is reported in Panel (**C**), for both CT26 (white columns) and 4T1 (black columns) cells, respectively. Only H6PD gene silencing (siRNA) significantly altered enzyme availability. By contrast, G6PD expression did not respond to any treatment (Panel **A**,**B**,**D**), confirming different regulatory mechanisms for the two enzymes. Enzymatic activity (expressed as mU x mg^−1^ of proteins) displayed an obviously different response, since MTF, the indirect H6PD inhibitor carbenoxolone (CBX), and gene silencing significantly decreased H6PD function (Panel **E**) without altering the G6PD one (Panel **F**). Again, the effect was extremely similar for both CT26 (white columns) and 4T1 (black columns) lines. Panels (**G**,**H**) display the effect the different treatments on FDG uptake (pink columns) and glucose consumption (green columns) in the same cell lines. Scramble administration was virtually ineffective. On the contrary, glucose intake was increased by MTF while being reduced by both CBX and gene silencing by siRNA. This pattern faced a direct effect of all treatments that significantly reduced FDG uptake in the same cell cultures. These data thus document that H6PD triggered metabolism is fueled by glucose at high rate. * = p < 0.01; **p < 0.001 vs corresponding control.

MTF did not alter the expression of both H6PD and G6PD (Fig. 3A–D). On the contrary, it selectively and markedly impaired H6PD catalytic function almost halving cell lysate capability to dehydrogenate 2DG6P in both cell lines (Fig. 3E) without affecting G6PD activity measured by G6P dehydrogenation rate (Fig. 3F).

### H6PD and cancer glucose metabolism

This finding provided a preliminary possible explanation of the paradoxical divergence of biguanide action on FDG uptake and glucose consumption, indicating that tracer retention might reflect the accumulation of trapped metabolites downstream of H6PD-catalyzed reaction. To test this hypothesis, we evaluated cell response to 24 hours exposure to the carbenoxolone (CBX)^33^ that inhibits H6PD function by decreasing NADP^+^ supply by 11-β−hydroxy-steroido-reductase^32^,^33^. CBX did not affect expression of both H6PD and G6PD (Fig. 3A–D). However, it significantly and selectively reduced cell lysates capability to dehydrogenate 2DG6P as an index of H6PD catalytic function (Fig. 3E), again without altering G6P dehydrogenation rate (Fig. 3F). These data are not surprising considering that the association between H6PD and CBX target 11-β−hydroxy-steroido-reductase^32^,^33^ was relatively preserved by our procedure that avoided the use of detergents in sample homogenization.

**Figure 4:**
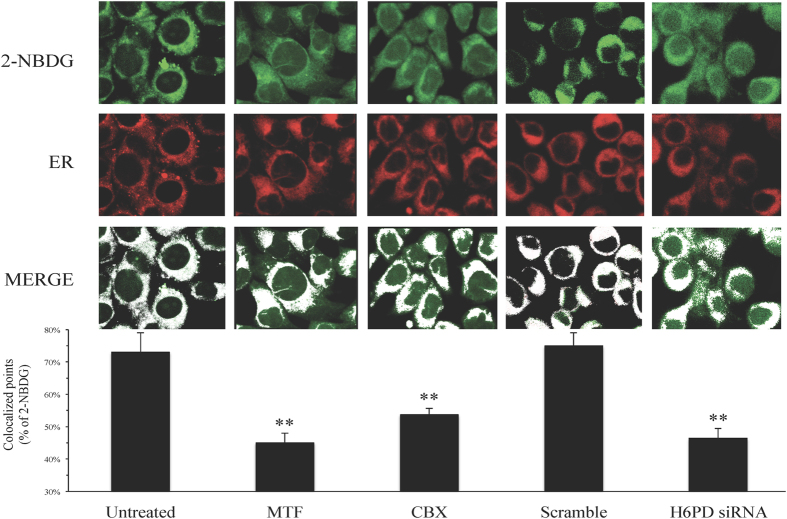
Glucose analogue 2-NBDG accumulates within the ER. Confocal microscopy images of 2-NBDG fluorescence (green) and of vital probe ER-TrackerTM Red (ER) in CT26 cells. Merge row represents the readout of imageJ processing reporting the colocalized signal as white pixels. Co-localization between the two probes was quantified on original images acquired under identical detection conditions. Nevertheless, since 2-NBDG uptake was markedly diminished in MTF, CBX and siRNA treated cells, the green fluorescence was enhanced in the reported images. In control (untreated) cells co-localization accounted for 73 ± 8% of 2-NBDG fluorescence as represented in the column chart at the bottom. ER entrapment of glucose analogue was markedly decreased by all treatments including 5 mM MTF, 180 μM CBX and siRNA (** = p < 0.01). By contrast, scramble-RNA was virtually ineffective. These data thus confirmed the major role played by ER in entrapment of glucose analogue.

More importantly, CBX significantly reduced total glucose consumption to 74 ± 3% and 62 ± 3% of control values in CT26 (Fig. 3G) and 4T1 (Fig. 3H) cells, respectively. Interestingly, FDG uptake at least partially reproduced this response and decreased under treatment to 63 ± 5% and 27 ± 2% of control values in the same cell lines (Fig. 3G,H).

Obviously, both MTF and CBX recognize a number of targets related to energy metabolism^4^,^5^,^36^ that might interfere with FDG retention and glucose consumption through a series of mechanisms independent of H6PD catalytic activity. To overcome this limitation, we selectively inhibited H6PD gene expression, transfecting both CT26 and 4T1 cells with short interfering RNA (siRNA) or SilencerTM Negative Control #1 siRNA (scramble). H6PD silencing was able to reduce cell lysate capability to dehydrogenate 2DG6P to 13 ± 0.7% and 22 ± 1% of control values, in CT26 and 4T1 cells, respectively (Fig. 3E). The specificity of this action was documented by the ineffectiveness of scramble administration (Fig. 3E) while the absent response of G6P dehydrogenation activity confirmed a completely different regulation of the cytosolic enzyme G6PD (Fig. 3F).

H6PD silencing “per se” largely reproduced CBX action on both metabolic indexes. In fact, glucose consumption – measured in the 24 hours after siRNA administration - was significantly reduced to 62 ± 3% and 60 ± 3% in CT26 and 4T1 cells, respectively (p < 0.001). More importantly, this effect paralleled the decrease in FDG uptake that fell to 73 ± 4% and 26 ± 2% of corresponding controls (p < 0.001), respectively (Fig. 3G,H).

Since the catalytic sites of both H6PD and G6Pase are located within the ER, the glucose analogues 2DG and FDG should selectively accumulate within this cell compartment. This issue is not trivial since the ionic nature of G6P and 2DG6P would indicate the possible involvement of specific carriers for trans-membrane transport^37^,^38^. Since affinity features and molecular characteristics of these proteins are largely unknown, we decided to verify whether 2DG6P actually accumulates within ER and whether the different treatments interfere with this localization. To this purpose, we used confocal microscopy to explore the intracellular distribution of the fluorescent 2DG analogue 2-NBDG^39^ and its co-localization with respect to vital ER probes. Image analysis with appropriate software tool (ImageJ, NIH) documented that co-localization of 2-NBDG and ER was high in untreated or “scramble” cells and was markedly reduced by all treatments (MTF, CBX or H6PD-siRNA) providing an image-based documentation of ER role in glucose metabolism and FDG uptake in CT26 and 4T1 cells (Fig. 4).

### H6PD and redox control of cancer biology

**Figure 5:**
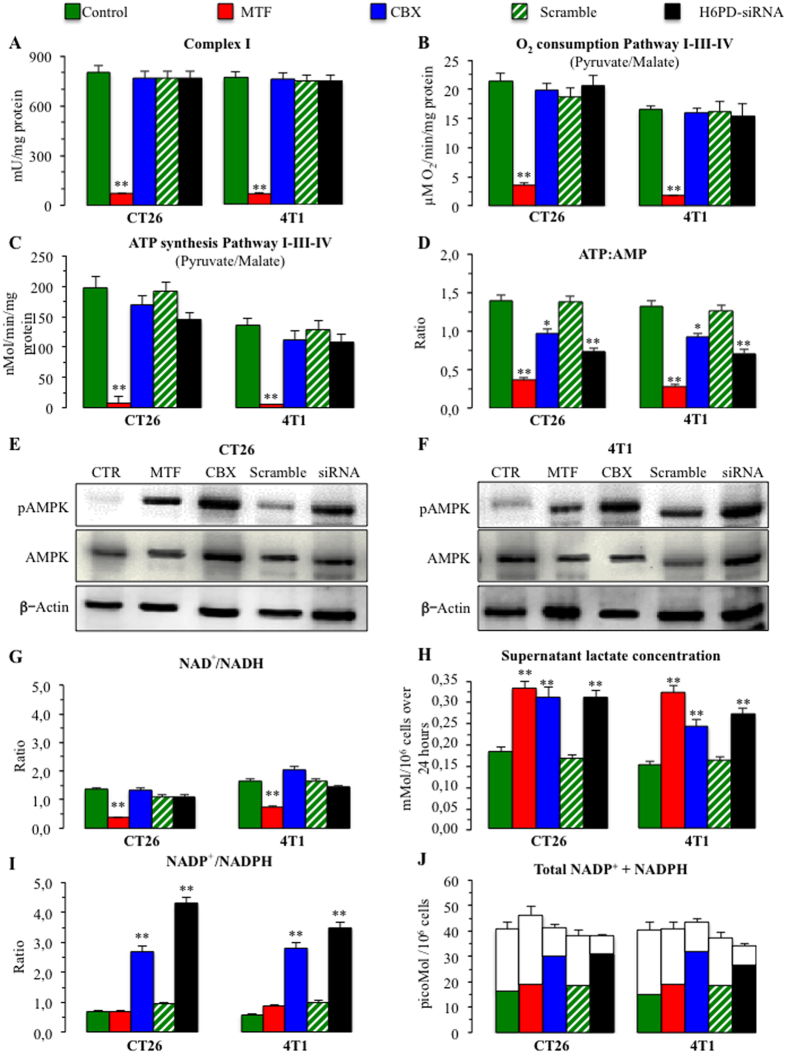
Cancer energy asset. Panel (**A**) represents the function of mitochondrial respiratory Complex I in CT26 and 4T1 cell lines, respectively. MTF virtually abolished Complex I activity (expressed as mU x mg^−1^ of proteins) that was instead left unaltered by all remaining treatments. This effect resulted in a marked decrease in the rate of both oxygen consumption (expressed as μMol O_2_ x min^−1^ x mg^−1^ or proteins in Panel (**B**) and ATP synthesis (expressed as nMol x min^−1^ x mg^−1^ or proteins in Panel (**C**) through the pathway I-III-IV interrogated by pyruvate-malate administration. Despite this markedly different effect on OXPHOS, ATP:AMP ratio was significantly decreased also by CBX and siRNA (Panel **D**) though to a lower degree with respect to MTF. Panels (**E**,**F**) display the original gels, run under the same experimental conditions for each cell line, documenting the expected response of the energy sensor mechanism that caused an increase in p-AMPK without altering total AMPK levels. The redox nature of H6PD triggered metabolism was confirmed by the decrease in NAD^+^ availability, since NAD^+^/NADH ratio was selectively decreased by MTF (panel **G**). By contrast, lactate release (expressed as mMol/10^6^ cells over 24 hours) was induced by all interventions but scramble (Panel **H**) despite an absent response of NADH levels. On the contrary, both CBX and siRNA, differently from the biguanide, increased the NADP^ + ^/NADPH ratio, without altering total coenzyme levels (measured in picoMol/10^6^ cells) (Panels **I**,**J**). (*=p < 0.05; ** = p < 0.01 vs corresponding controls).

Differently from MTF, both pharmacologic H6PD impairment by CBX and selective silencing of its expression decreased overall glucose consumption of studied cancer cells suggesting an energetic role for ER metabolism independent from OXPHOS. This hypothesis was strikingly confirmed. Actually, mitochondrial function was impaired by MTF that severely inhibited Complex I activity^4^,^5^ (Fig. 5A) causing a marked decrease in both oxygen consumption rate and ATP synthesis through pathway I-III-IV (Fig. 5B,C), despite a normal function of Complexes II-III-IV and their corresponding pathways (Supplementary Figure 5A–C). However, both CBX and siRNA significantly reduced total ATP asset (Supplementary Figure 6A) and ATP:AMP ratio (Fig. 5D) without hampering mitochondrial OXPHOS (Fig. 5A–C, Supplementary Figure 5D,E). Degree of energy depletion caused by H6PD inhibition was actually less evident with respect to MTF. Nevertheless, its relevance was confirmed by the activation of the sensor mechanism AMP-activated protein kinase (AMPK)^40^ whose phosphorylation was comparably enhanced by all treatments (Fig. 5E,F, Supplementary Figure 6B,C).

The mitochondrion-independent and redox nature of ER glucose catabolism was corroborated by the analysis of the pyridine dinucleotides. In fact, NAD^+^/NADH ratio only *decreased* under MTF (Fig. 5G, Supplementary Figure 7). This response most likely reflected Complex I inhibition and resulted in an accelerated glycolytic flux with a consequent increase in lactate release (Fig. 5H). On the contrary, and in line with the expected response to H6PD inhibition, both CBX and siRNA selectively *increased* NADP^+^/NADPH ratio (Fig. 5I,J) confirming the role of this pathway in keeping ER redox balance in a reduced state. Moreover, despite a preserved NAD^+^ availability, both treatments significantly increased lactate release, indicating that complete glucose oxidation is at least partially independent from mitochondrial function (Fig. 5H).

**Figure 6:**
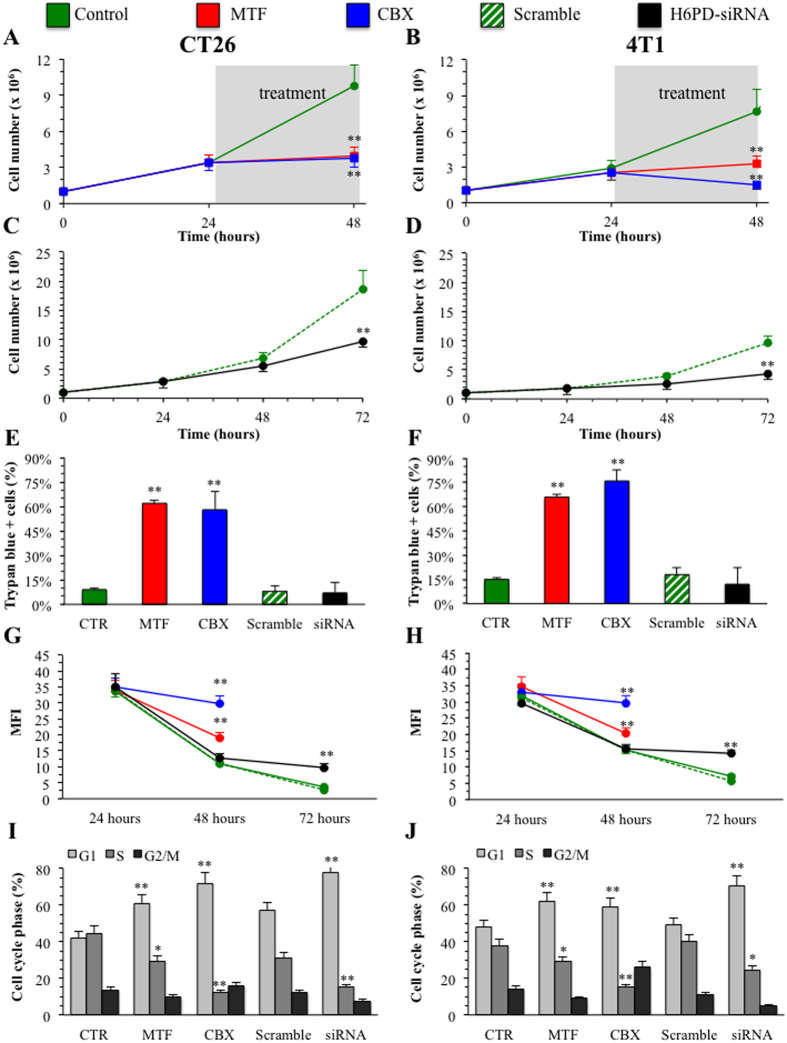
Cancer biological response to energy depletion. Growth of both cell cultures was impaired by 24 hours exposure to 5 mM MTF (red line) and CBX (blue line, at 180 μM for CT26 and 135 μM for 4T1) (Panel **A**,**B**) lines, with respect to untreated cell (green line). A similar, though delayed, effect was observed under H6PD gene silencing (black line) but not under scramble administration (green hatched line) in both CT26 (Panel **C**) and 4T1 (Panel **D**) lines. Cell growth reflected the contribution of cytotoxic and/or cytostatic effects of the different treatments. Viability analysis with Trypan blue (Panel **E,F**) demonstrated a significant increase in cell death caused by MTF (red columns) and CBX (blue columns) treatment (**=p < 0.01), while gene silencing (black columns) left virtually unaltered cell viability with respect to untreated (green columns) and scrambled (green hatched columns) conditions. By contrast, all treatments affected cell proliferation rate. In fact, CFSE analysis on the same cell cultures documented that mean fluorescence intensity (MFI), which is inversely related to cell proliferation, was lowered by all interventions (Panels **G**,**H**). This finding was confirmed by cell cycle analysis that documented a block in G1 phase under all treatments (Panel **I**,**J**). (*=p < 0.05; **=p < 0.01 statistical differences vs corresponding controls).

The fundamental role of H6PD-triggered glucose catabolism in cancer cell survival and growth was confirmed by the effect of enzyme interference on cell viability and proliferation rate. Actually, all treatments flattened growth curves of cell cultures (Fig. 6A–D). However, while siRNA selectively decreased proliferation rate, both MTF and CBX combined this cytostatic action with a significant cytotoxic effect (Fig. 6E–H). Finally, this biological response closely agreed with the analysis of cell cycle distribution that displayed an evident block in G1 phase under all treatments (Fig. 6I,J).

## Discussion

The divergent effect of MTF on glucose consumption and FDG uptake in cancer cells brought us to discover a still unidentified pathway for glucose utilization, largely autonomous from the well-recognized cytosolic glycolysis and PPP. This conclusion is first based on the evidence that retention of both FDG and 2DG reflects the activity of an ER processing machinery in which H6PD competes with G6Pase to process phosphorylated monosaccharides. This pathway contributes to ER redox balance by preserving a low NADP^+^/NADPH ratio. However, it also contributes to maintain the whole cell energy asset since its inhibition decreases the ATP:AMP ratio and activates AMPK phosphorylation. Coherently with this role, H6PD-dependent pathway is fueled by glucose at rates approaching cytosolic carbohydrate utilization, since H6PD silencing results in a well measurable decrease in overall glucose consumption. These observations thus extend H6PD function far beyond its acknowledged role in signaling pathways related to NADPH-dependent activation of steroid hormones^30^,^31^,^32^,^37^.

So far, all current models of energy metabolism consider glucose consumption a well-established process managed by glycolysis and PPP. These data document, after more than 70 years, the existence of a further mechanism that, although still qualitatively uncharacterized, displays an activity close to the classical pathways. Moreover, both selective inhibition of H6PD enzyme activity and MTF similarly increased lactate release despite divergent effects on glucose consumption and, mostly, on NAD^+^ availability. These data thus indicate that ER metabolism contributes to the full conversion of glucose to water and CO_2_, at least in cancer cells. Together with the proteomic evidence of OXPHOS proteins within ER^41^, this finding suggests that control of ER redox state might be at least partially independent from mitochondrial function with both compartments contributing to cell energy asset through different pathways for glucose catabolism. The present data do not elucidate whether and how ER processing machine interacts with the most recognized cytosolic processes glycolysis and PPP. Similarly, they do not identify the mechanisms underlying its regulation. Finally, they do not clarify whether this metabolic pattern is universal or specific in cancer. To this purpose dedicated experiments would be needed to account for the effect of peculiar composition of culture media needed to warrant an adequate growth of “normal” cell cultures. Nevertheless, the innovative nature of our findings challenges the currently accepted Warburg hypothesis of an accelerated glycolytic flux as the link between high glucose consumption and cell proliferation in cancer1.

These findings represent a paradigm shift in energy metabolism whose implications in cancer biology are far beyond our scopes. However, the present data “per se” display a great clinical relevance in the daily practice asking for PET/CT imaging in cancer patients. The model used in this setting has been mostly derived from studies on central nervous system while its application in normal tissues of different origin already asked for heuristic normalization factors, usually defined as “Lumped constant”, i.e. the ratio between FDG accumulation and glucose intake^42^,^43^,^44^. This factor is commonly attributed to the peculiar substrate affinity pattern of the different isoforms of HK and GLUT^45^ in the different tissues or under different conditions. The obligatory role of H6PD function and the lack of proportionality between tracer uptake and glucose consumption under MTF provide a new, alternative, explanation for the well-recognized direct relationship between FDG accumulation and cancer aggressiveness^46^,^47^. In this line, FDG uptake might be an index of H6PD activity and its link with cell proliferation more than a surrogate index of glycolysis rate in growing cancer. This novel clue has the potential to improve our comprehension of cancer evolution in patients and might represent the basis for a novel approach to explain the variability of lesion tracer uptake, in particular in those cancers usually unable to retain FDG despite a high glycolytic flux.

## Materials and Methods

### Chemicals

MTF was provided by Sigma-Aldrich (St. Louis, MO, USA). CBX was provided by Abcam (Cambridge, UK). FDG was produced according to standard methodology. Daily quality controls always documented a radiochemical purity ≥98%.

### Cell lines and culture conditions

The murine cancer cell lines CT26 (colon) and 4T1 (breast) and the human cancer cell lines Calu-1 (lung), MDA-MB231 (breast), LNCaP (prostate) and IMR-32 (neuroblastoma) were purchased from ATCC (LGC Standards Srl, Milan, Italy). The human melanoma cell line LB24Dagi (LB24) was kindly provided by Dr. D. Castiglia (Istituto Dermopatico dell’Immacolata, Department of Molecular and Cellular Biology, Rome). All cancer cell lines were cultured at 37 ^o^C under 5% CO_2_ in DMEM medium (Euroclone, Milan, Italy) supplemented with 1% L-glutamine, penicillin/streptomycin, nonessential amino acids and 10% fetal bovine serum (all from Sigma Aldrich). In all cases experiments were performed in triplicate, glucose concentration of administered medium was set at 11.1 mM. MTF treatments were performed for 24 h at concentrations ranging from 1 mM to 10 mM, while CBX was administered at 180 μM for CT26 and at 135 μM for 4T1 on the basis of preliminary assessment of IC50 dose.

### Animal Models

All animal experiments were performed in accordance with guidelines and regulations (Italian 26/2014 and EU 2010/63/UE directives) and were thus approved by the Licensing and Ethical Committee of IRCCS San Martino-IST and by the Italian Ministry of Health. Six- weeks-old female BALB/c mice were purchased from Charles River Laboratories (Lecco, Italy) and housed under specific pathogen-free conditions. Animals were inoculated subcutaneously in the dorsal hip with 2 × 10^5^ CT26 cells or 4T1cells. Each group was subsequently divided into four groups (two for each cell lines): “control” group (n = 7) and “MTF” group (n = 7). MTF was orally administered by diluting in autoclaved drinking water at a concentration of 3 mg/mL accounting for a dose of 750 mg/Kg/die^8^,^18^. Experimental protocol implied two imaging studies, at day #7 and day #14 from cell injection. Before each PET scan, mice were kept under fasting conditions for 6 h with free access to water. Cancer volume was determined by using external caliper and tumor volume was calculated using the following equation: tumor volume (mm3) = (length × width × height) × π/6, expressing length, width and height in mm. Mice were euthanized by CO_2_ asphyxiation at the end of experiments.

### Experimental micro-PET scanning protocol

*In vivo* imaging was performed according to a protocol validated in our lab^8^,^18^. Mice were weighted and anesthesia was induced by intra-peritoneal administration of ketamine (100 mg/Kg) (Imalgere 1000, Milan, Italy)/xylazine (10 mg/kg) (Bio98 Srl, Milan, Italy). Serum glucose level was tested and animals were positioned on the bed of a dedicated micro-PET system (Albira, Carestream Inc, Rochester, MN, USA) whose two-ring configuration permits to cover the whole animal body in a single bed position. A dose of 3–4 MBq of FDG was then injected through a tail vein, soon after start of a list mode acquisition lasting 50 minutes.

### Image processing

Acquisition was reconstructed using the following framing rate: 10 × 15 s, 5 × 30 s, 2 × 150 s, 6 × 300 s, 1 × 600 s. PET data were reconstructed using a maximal likelihood expectation maximization method (MLEM). An experienced observer, unaware of the experimental type of analyzed mouse, identified a volume of interest (VOI) in the left ventricular chamber. Then, the computer was asked to plot the time-concentration curve within this VOI throughout the whole acquisition to define tracer input function. Further VOIs were drawn over cancer lesions to measure average metabolic rate of glucose per unit mass (MRGlu) was expressed in nM X min^−1^ X g^−1^; total lesion consumption was provided by the product MRGlu X (lesion volume) and was estimated in these last VOIs according to Gjedde-Patlak^19^ graphical analysis by using the routine of a dedicated software (PMOD, Zurich, Switzerland). Briefly, the software utilizes the input function to transform the original tissue activity measurements by fitting the data in each voxel with the slope of the regression line defined by the model. In all cases, lumped constant value was set at 1.

### Spectrophotometric Analysis

Glucose consumption was evaluated by monitoring supernatant concentration throughout the 24 hours preceding the measurement of FDG uptake according to standard procedure^48^.

Enzymes activities were assayed on 50 μg of total proteins as previously described^7^. Briefly, HK activity was estimated spectrophotometrically by following NADP at 340 nm, through a coupled reaction with G6PD. G6Pase activity was measured at 660 nm, following the inorganic phosphate production by the Fiske and Subbarow method. Capabilities to dehydrogenate G6P and 2DG6P (as indexes of G6PD and H6PD function, respectively) were evaluated in cell homogenates obtained with two different methods. In the former case, cells were sonicated two times for 10 s, with an interval of 30 s in ice; in the latter, cells were sonicated twice for 20 s, maintaining an interval of 30 s in ice. Detergents were never used in cell lysate preparation.

The medium lactate concentration was determined following the reduction of NAD^+^ at 340 nm. In all cases, metabolite absence in culture medium was tested^48^. The intracellular concentrations of ATP and AMP were measured, following the reduction of NADP and the oxidation of NADH, respectively, as previously described^49^.

### FDG Uptake Evaluation

In all cases *in vitro* experiments were performed in triplicate. Cell culture was maintained at 37 °C for 60 minutes in a glucose free medium with FDG concentration set at 370 KBq/mL FDG, then the supernatant was collected and cells were recovered. Free and bound activities were thus simultaneously counted using a Packard Cobra II gamma counter (Packard, Meriden, CT, USA) with a 10% energy window centered at 511KeV. FDG retention was measured as the ratio between bound and total radioactivity. Labeling procedure did not affect cell viability.

### Western blot analysis

Western blot experiments were performed accordingly to the standard procedure with the following antibodies: anti-GLUT1, anti-HKII, anti-Phospho-AMPK (Cell Signaling, Danvers, MA, USA), anti-G6PD (Sigma Aldrich), anti G6Pase, anti-H6PD (Abcam) and anti-β-actin (Santa Cruz Biotechnology, Dallas, TX, USA).

### Co-localization experiments

Intracellular localization of HKII was studied on cells cultured on glass coverslips and treated with MitoTracker probe (Life Technologies Ltd, Monza MB, Italy), rabbit anti-HKII (C64G5) primary antibodies (Euroclone) and then with a goat anti-Rabbit Alexa Fluor 488 secondary antibody (Molecular Probes Eugene, OR, USA). Results were analyzed using an Olympus (Olympus Optical) laser-scanning microscope FV500 equipped with an Olympus IX81 inverted microscope and Argon ion 488 nm, He-Ne 543 nm, and He-Ne 633 nm lasers. Digital images were acquired through a PLAPO 60× objective, with the Fluoview 4.3b software program. Images were acquired sequentially as single trans-cellular optical sections. Spatial co-localization was analyzed by Image J 1.34f software (NIH).

Glucose metabolite distribution and co-localization with the endoplasmic reticulum (ER) were evaluated by confocal microscopy on live cells grown in glass-bottom dishes, stained for 15 minutes with the fluorescent probes 2-[N-(7-nitrobenz-2-oxa-1,3-diazol-4-yl)amino]-2-deoxyglucose (2-NBDG) (50 microM) and ER-Tracker^TM^ Red (1 microM), both from Molecular Probes (InVitrogen, Eugene, OR), and analyzed by the SP2-AOBS confocal microscope (Leica Microsystems, Mannheim, Germany). Six to eight randomly selected fields containing at least 8–10 cells were analysed in three independent samples for each treatment. Original unadjusted and uncorrected images were processed by ImageJ algorithms for the evaluation of colocalization, which was expressed as the percentage of above-background pixels in 2-NBDG images that overlapped above-background pixels in ER images, with background threshold set by the Costes’ method.

### Oxygen consumption and ATP synthase assay

O2 consumption was measured at 25 °C in a closed chamber (1.7 ml capacity) using a thermostatically controlled oxygraph apparatus equipped with amperometric electrode (Microrespiration, Unisense A/S, Århus, Denmark) as previously described^50^.

ATP concentration was measured in a luminometer (Lumi-Scint, Bioscan) by the luciferin/luciferase chemiluminescent method^48^. In both cases, 5 mM pyruvate +2,5 mM malate or 20 mM succinate were used as respiring substrates to assess the activity of the pathway formed by Complexes I, III and IV and the pathway composed by Complex II, III and IV, respectively.

### Respiratory Complexes assay

The activity of the four respiring complexes was assayed on 50 μg of total protein. Complex I (NADH-ubiquinone oxidoreductase) was assayed following the reduction of ferricyanide at 420 nm^50^.

Complex II (Succinic dehydrogenase) activity was measured at 600 nm, in 2 mM EDTA, 0.2 mM ATP, 20 mM succinate, 0.5 mM cyanide, 80 μM dicloroindophenol (DCIP), 50 μM decylubiquinone, 40 μM antimycin A, 10 μM rotenone and 10 mM phosphate buffer, pH 7.2^50^.

To measure Complex III (Cytochrome c reductase) activity was followed the reduction of oxidized Cytochrome c, at 550 nm^21^. Finally, Complex IV (Cytochrome c oxidase) was assayed following the oxidation of ascorbate-reduced Cytochrome c at 550 nm^50^.

### NAD^+^/NADH and NADP/NADPH determination

The ratio between NAD^+^:NADH and NADP:NADPH in cell lysates were evaluated spectrophotometrically, at 450 nm, using the NAD/NADH Assay Kit (Abcam: ab65348) and NADP/NADPH Assay Kit (Abcam: ab65349), respectively following the manufacture’s instructions.

### Transfection assay

Silencing of H6PD expression was achieved by transfecting cells with H6PDH siRNA (Ambion siRNA ID 14371) or Silencer^TM^ Negative Control #1 siRNA (Ambion, Huntingdon, Cambs, UK) both 40 pMoles/ml, using Lipofectamine 2000 as transfection agent (2.5 microL/mL).

### Cell viability, proliferation and cell cycle analysis

Cell viability was evaluated by Trypan blue (Sigma Aldrich) exclusion test. To asses proliferation, cancer cells were labeled with 20 μM CarboxyfluoresceinSuccinimidyl ester (CFSE) (InVitrogen) following the manufacture’s instructions. Samples were acquired in a Gallios cytometer (Beckman Coulter Spa, Cassina de’ Pecchi , MI) and analysed using Kaluza software. The percent of cells in G1, S and G2/M was determined by flow cytometer. Briefly cells were fixed in 80% ethanol, washed in PBS and stained with PI/RNase buffer. The acquisition and analysis were performed as previously described^49^.

### Statistical analysis

The data are presented as mean ± standard deviation (SD). For comparison between different groups, the Null hypothesis was tested by analysis of variance (ANOVA) for multiple comparison; it was tested by Student *t* test for paired or unpaired data, as appropriate. Statistical significance was considered for p values p < 0.05. Statistical analyses were performed using SPSS software Advanced Models 15.0 (Chicago, Illinois).

## Additional Information

**How to cite this article**: Marini, C. *et al*. Discovery of a novel glucose metabolism in cancer: The role of endoplasmic reticulum beyond glycolysis and pentose phosphate shunt. *Sci. Rep.* **6**, 25092; doi: 10.1038/srep25092 (2016).

## Supplementary Material

Supplementary Information
