# Supplementary material for: Discovery of a novel glucose metabolism in cancer: The role of endoplasmic reticulum beyond glycolysis and pentose phosphate shunt
Source: Sci Rep. 2016 Apr 28;6:25092. doi: 10.1038/srep25092 (PMC4848551; doi:10.1038/srep25092)
Supplement: Supplementary Information [file srep25092-s1.pdf]

Discovery of a novel glucose metabolism in cancer: The role of endoplasmic reticulum beyond glycolysis and pentose phosphate shunt.

Cecilia Marini[1,2*], Silvia Ravera[3*], Ambra Buschiazzo[2], Giovanna Bianchi[4], Anna Maria Orengo[2], Silvia Bruno[5], Gianluca Bottoni[2]; Laura Emionite[6]; Fabio Pastorino[4]; Elena Monteverde[2]; Lucia Garaboldi[2], Roberto Martella[4]; Barbara Salani[7]; Davide Maggi[7]; Mirco Ponzoni[4]; Franco Fais[5,8], Lizzia Raffaghello[4]; Gianmario Sambuceti[2*].

*All these authors acted as first author

[1] CNR Institute of Molecular Bioimaging and Physiology (IBFM), Milan, Section of Genoa, Genoa, Italy
[2] Nuclear Medicine Unit, Department of Health Sciences, University of Genoa and IRCCS AOU San Martino-IST, Genoa, Italy
[3] Stem Cell Center, IRCCS G. Gaslini, Genoa, Italy
[4] Laboratorio di Oncologia, IRCCS G. Gaslini, Genoa, Italy
[5] Department of Experimental Medicine, University of Genoa, Genoa, Italy
[6] Animal facility, IRCCS AOU San Martino-IST, Genoa, Italy
[7] Department of Internal Medicine, University of Genoa and IRCCS AOU San Martino-IST, Genoa, Italy
[8] Molecular Pathology, IRCCS AOU San Martino-IST, Genoa, Italy

Running title: *Discovery of a novel glucose metabolism in cancer.*

Keywords: cancer, glucose metabolism, FDG, PET, hexose-6-phosphate dehydrogenase.

Address for correspondence:
Gianmario Sambuceti, MD,
C/o Nuclear Medicine
IRCCS AOU San Martino-IST,
16132- Genoa,
Italy
Email: Sambuceti@unige.it

Total word count (from introduction to end of discussion): 2905
Number of Figures: 6

Suppl. Table 1: Body weight (in grams) and serum glucose level (in mmol/l) in each study group at PET scan time (day 7) and after 14 days from tumor implantation

		**Weight (grams)**		**Serum glucose (mmol/L)**	
		**Day 7**	**Day 14**	**Day 7**	**Day 14**
**CTR**	CT26 (n=7)	18,60 ± 1,48	17,71 ± 1,23	4,26 ± 1,22	3,50 ± 2,59
	4T1 (n=7)	16,17 ± 0,75	17,55 ± 0,56	4,71 ± 0,74	4,74 ± 0,88
**MTF**	CT26 (n=7)	15,91 ± 0,61	17,31 ± 1,05	4,24 ± 1,42	3,50 ± 1,21
	4T1 (n=7)	16,36 ± 0,78	17,66 ± 0,78	4,40 ± 1,20	5,14 ± 1,48

Suppl. Figure #1

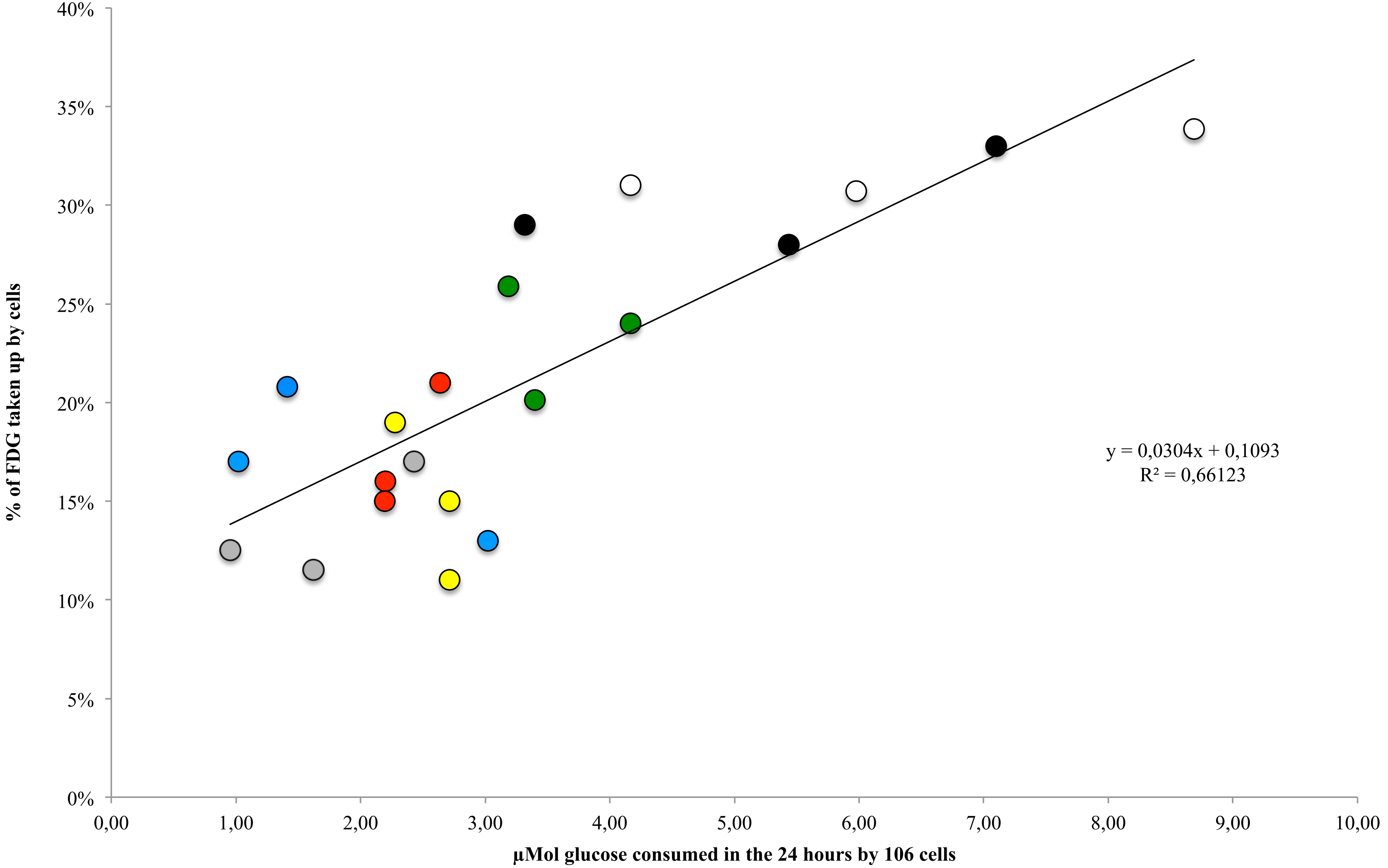

Suppl. Fig. 1: Scatterplot of glucose consumption over 24 hours (in microMol per million cells on X axis) and % FDG uptake over one hour per million cells (Y axis). The seven cell lines (4T1-black, CT26-white, Calu-1-green, MDA-MB231-red, LNCAP-gray, GI-LI-N-blue and LB24-yellow as models of murine breast carcinoma, murine colon cancer, human non small cell lung cancer, human triple negative breast cancer, human prostate cancer, human neuroblastoma and melanoma) were studied in triplicate and all 21 points are reported. The experiments confirmed the expected correlation between the two metabolic indexes in the absence of any treatment.

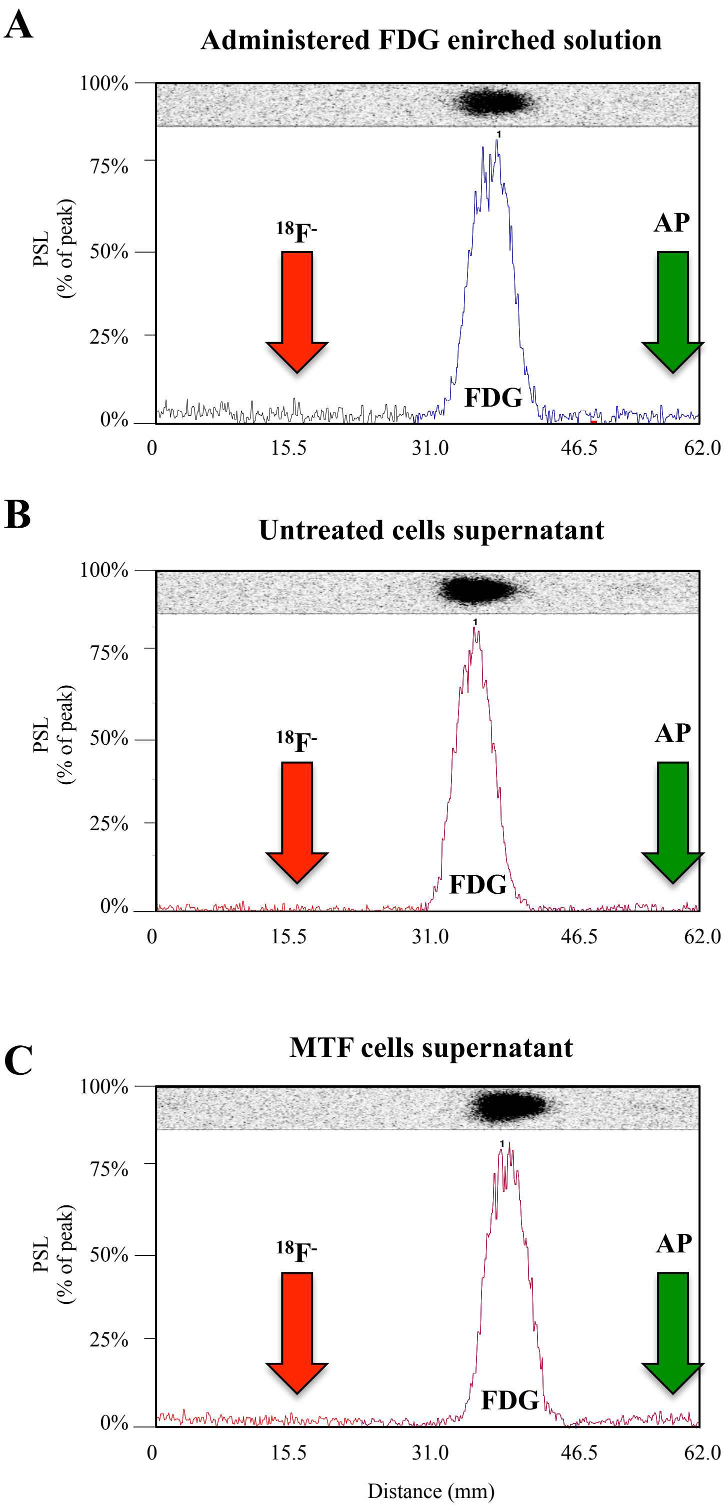

Suppl. Fig. 2: Original results of thin layer cromatography obtained in administered medium (A) as well in supernatant collected 60 minutes after exposure to control cells (B) or MTF-treated cells (C). A ten centimeters long silica gel thin chromatography paper (Merk, Germany) was used. A small mark was placed on one end and a small drop of ^{18}F-FDG (1µL) was put on TLC strip. The TLC was put into a TLC developing jar containing developing solution (95:5 acetonitrile: water V/V). When the developing solution reached more than eight centimeters on the TLC strip, it was removed and dried. Thereafter, TLC scanning was started for results. Virtually no $^{18}F^-$ was detectable under any condition (red arrows). Similarly, no acetylated products (AP, green arrows) could be observed while virtually the whole amount of radioactivity was detected in the FDG area.

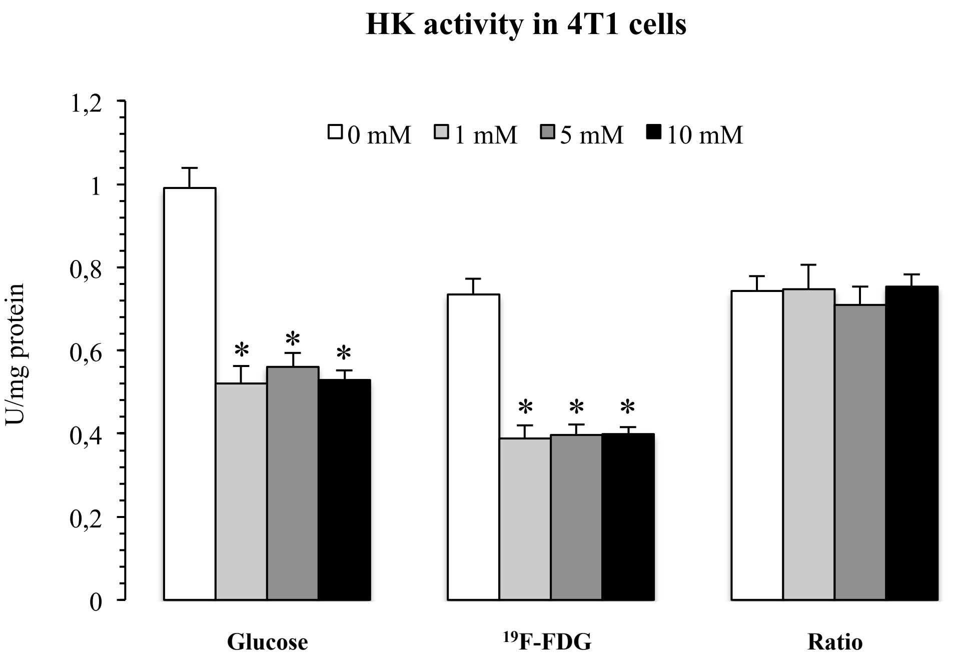

Suppl. Fig. 3: The moderate decrease of glucose phosphorylation rate of cell lysates of 4T1 cells (expressed as U x mg^{-1} of proteins). However, this reduction in overall HK activity could not explain the divergent responses of glucose and FDG to MTF. Similarly to CT26, cell lysate affinity ratio for glucose and nonradioactive FDG form ^{19}F-FDG remained stable despite increasing drug concentrations.

Suppl. Figure #4

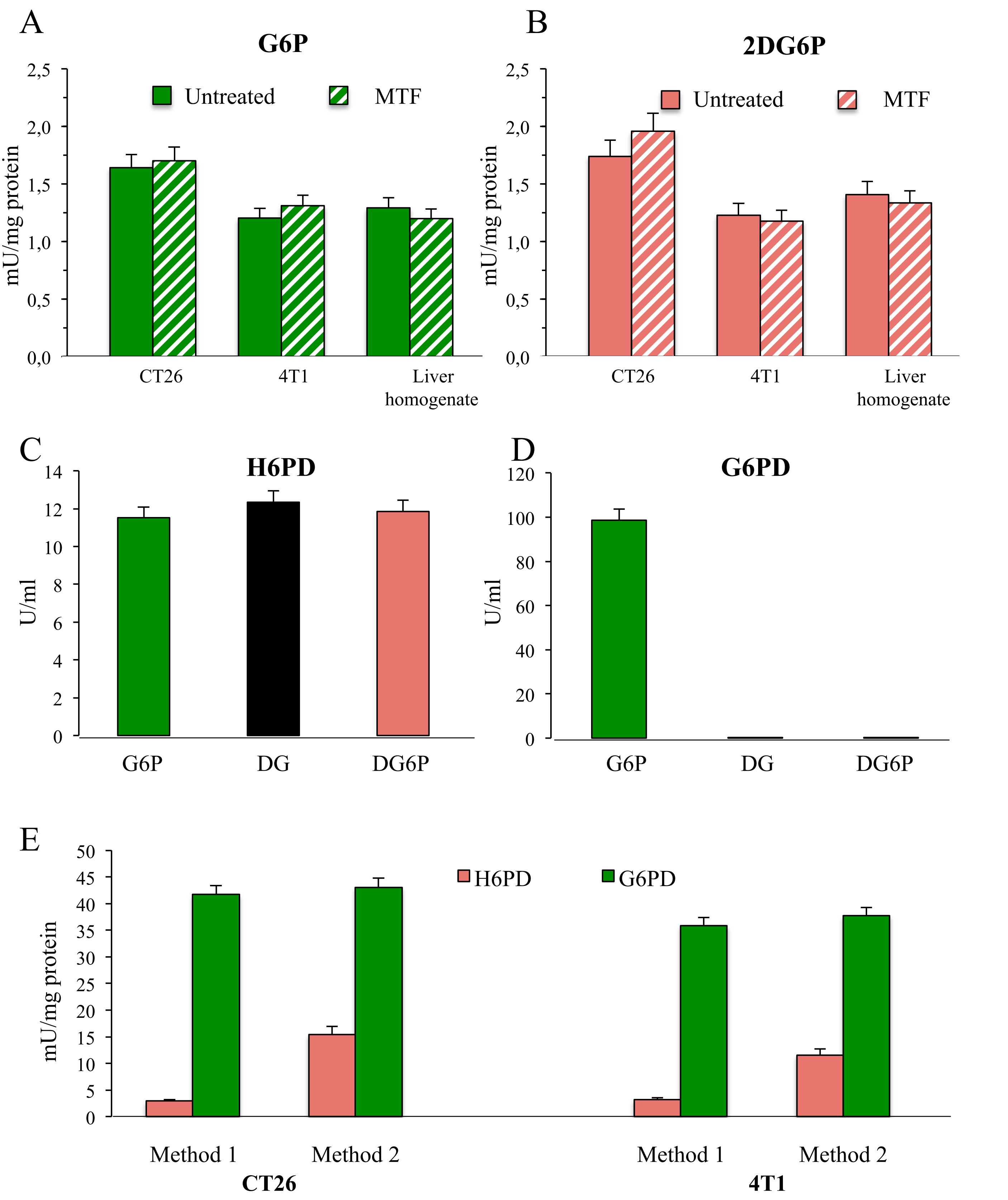

Suppl. Fig. 4: Cell lysate G6Pase was similarly active in dephosphorylating G6P (A) and 2DG6P (B) in both CT26 and 4T1 cell lines. Addition of MTF (dashed columns) to the reaction medium did not affect enzyme activity. Finally, G6Pase activity was similar in studied cancer cells and in rat liver homogenate. C and D: Enzyme activity of purified H6PD (C) and G6PD (D) on different substrates represented by G6P (green columns), 2DG (Black columns) and 2DG6P (pink columns). G6PD was active only on G6P while H6PD showed a similar affinity for the three substrates. A large difference in G6PD dehiydrogenation rate was observed with G6PD markedly more active than H6PD. Panel E: Dehydrogenation rate of 2DG6P (pink columns) and of G6P (green columns) in cell lysates exposed to 10 sec (method 1) or 20 sec (method 2) periods of sonication with an interval of 30 sec in ice. Different procedures resulted in different extraction of H6PD activity without altering overall G6PD function.

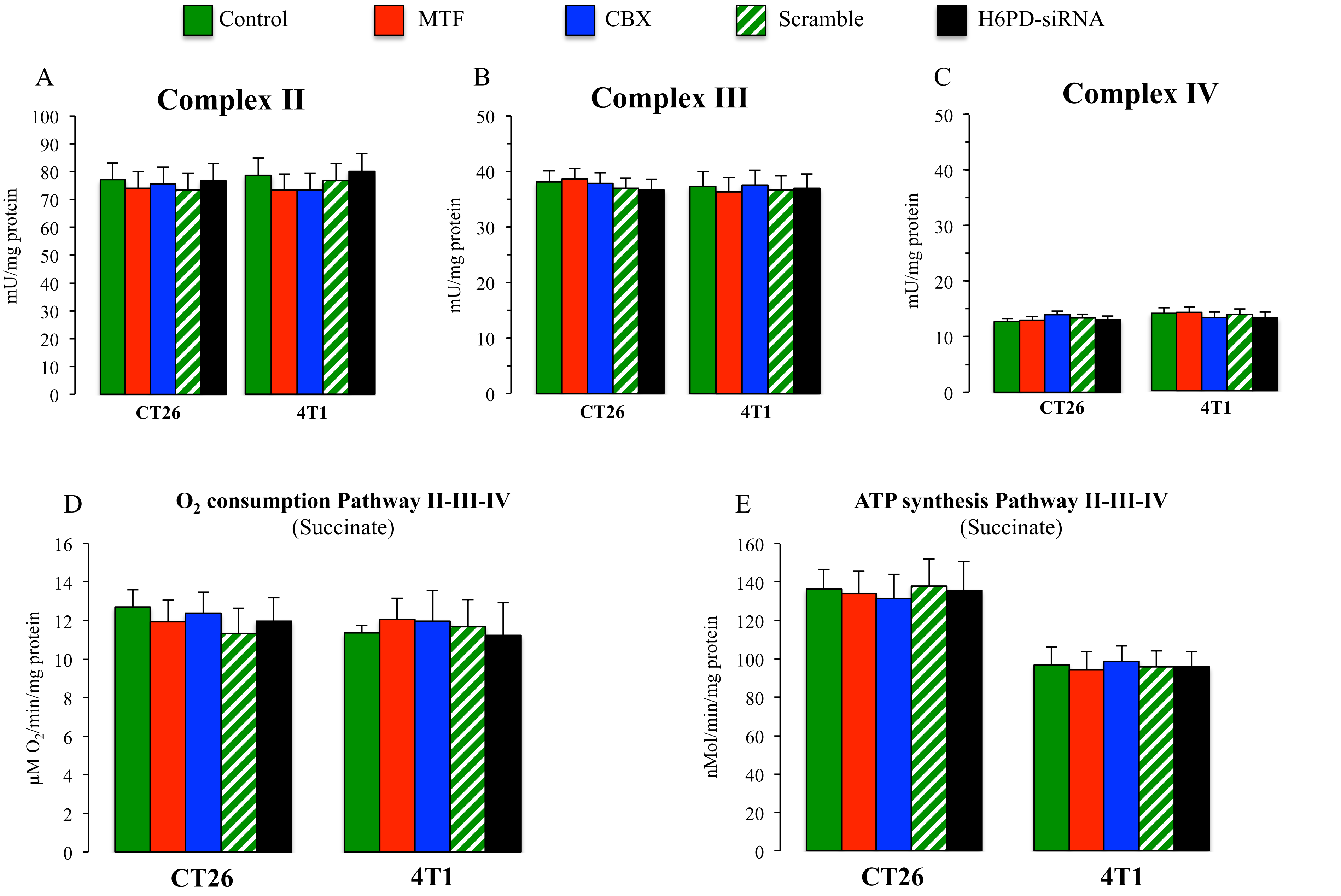

Suppl. Fig. 5: Panels A-C display the absent response of mitochondrial II-III-IV respiratory complexes. Panel D-E report oxygen consumption rate and ATP synthesis rate for pathway II-III-IV.

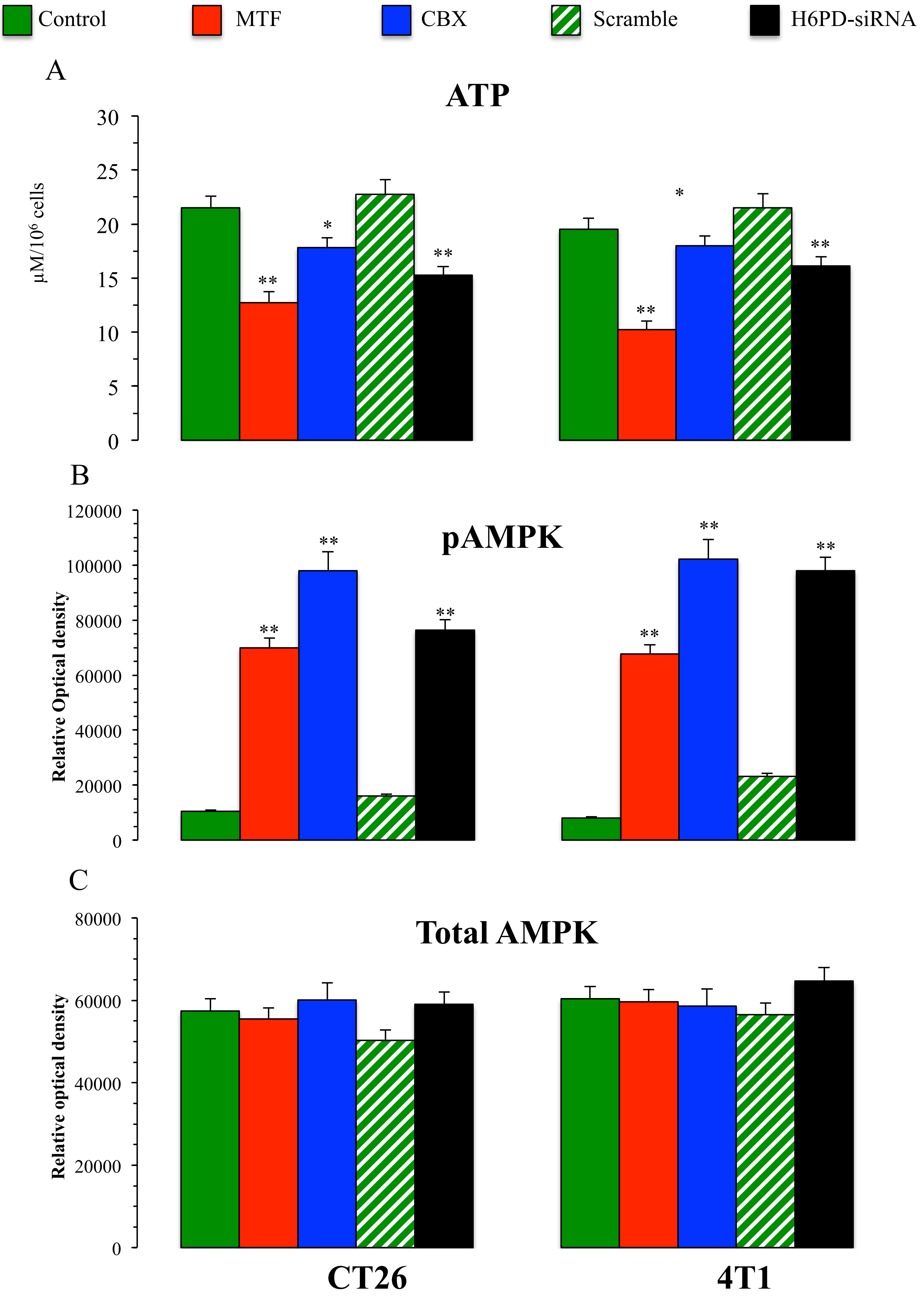

Suppl. Fig. 6: Panel A displays total ATP asset in CT26 and 4T1 cells that was decreased by all treatments but scramble. This response was paralleled by the marked increase in AMPK phosphorylation as documented by pAMPK (panel B) and total AMPK (panel C) levels (*=p<0.01; **=p<0.001).

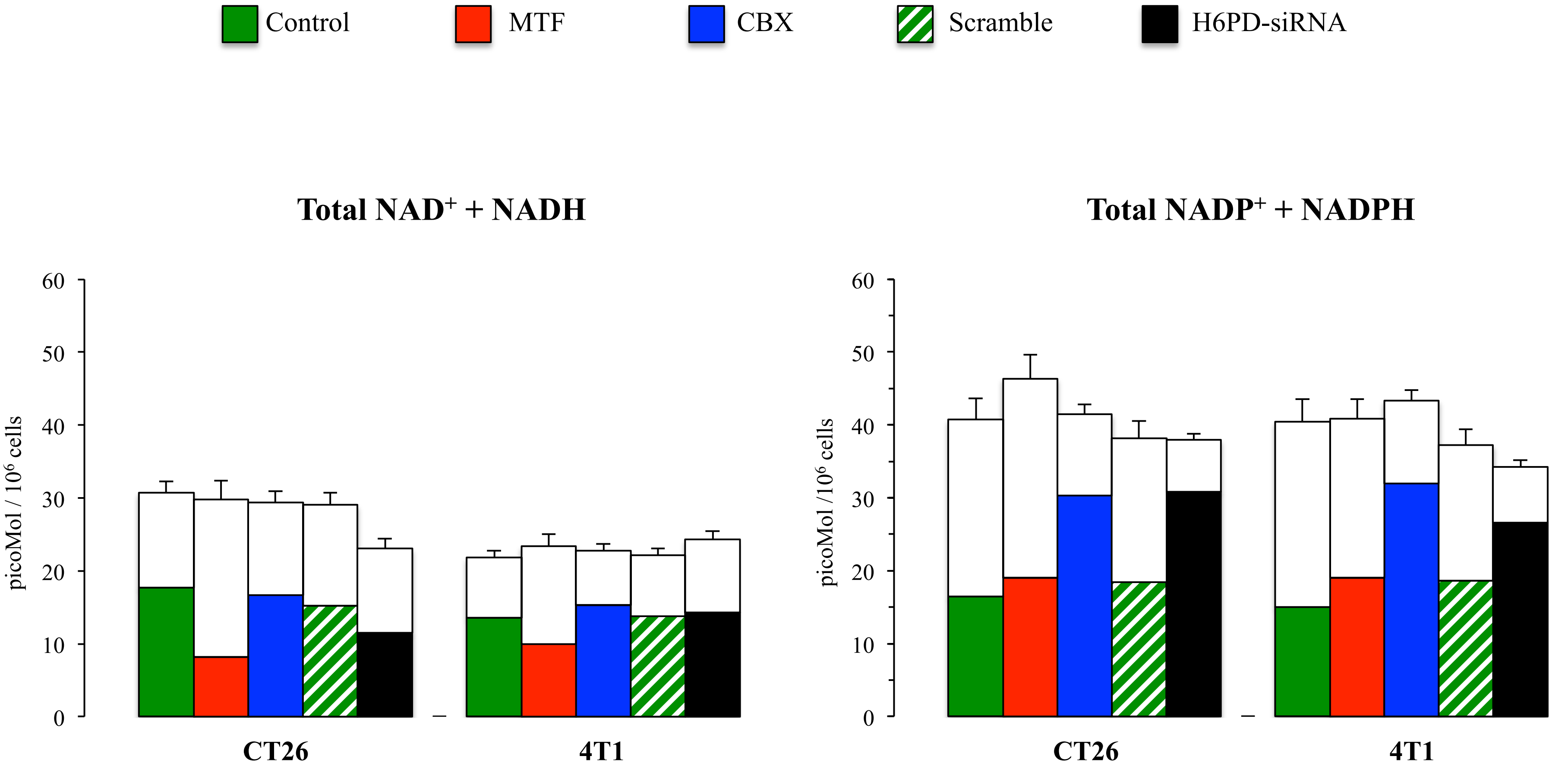

Suppl. Fig. 7: Total availabilities of Nicotinamide adenine dinucleotide (NAD) and Nicotinamide adenine dinucleotide phosphate (NADP) in CT26 and 4T1 cells (represented according to the color code conventionally used throughout the paper) were left virtually unchanged by all treatments. Colored columns represent the oxidized form while the reduced one is represented in white over the corresponding treatment. MTF exposure caused a selective decrease in oxidized NAD^+ concentration due to the inhibition of Complex I activity. By contrast, both CBX and siRNA caused a selective increase in $NADP^+$ levels.
